# Luteolin mitigates proliferative vitreoretinopathy through inhibition of ERK1/2 signaling and epithelial–mesenchymal transition

**DOI:** 10.3389/fphar.2026.1634617

**Published:** 2026-02-03

**Authors:** Lingdan Wu, Meiling Chen, Jinghua Chai, Yujie Tang, Qihua Xu

**Affiliations:** 1 Nanchang University, Nanchang, Jiangxi, China; 2 Jiangxi Research Institute of Ophthalmology and Visual Sciences, Nanchang, Jiangxi, China; 3 Department of Ophthalmology, The Affiliated Eye Hospital, Jiangxi Medical College, Nanchang University, Nanchang, Jiangxi, China; 4 Department of Ophthalmology, The People’s Hospital of Longhua, Shenzhen, Guangdong, China

**Keywords:** proliferative vitreoretinopathy, epithelial-mesenchymal transition, retinal pigment epithelial cell, luteolin, TGF-β2

## Abstract

**Aim:**

To investigate the inhibitory effect of luteolin on proliferative vitreoretinopathy (PVR) and explore its potential mechanism.

**Methods:**

Human retinal pigment epithelial ARPE-19 cells were treated with luteolin (0–50 μM) to assess its effects on cell viability, migration, and TGF-β2–induced epithelial–mesenchymal transition (EMT). Cell viability (CCK-8), Scratch and Transwell assays, flow cytometric cell-cycle analysis, immunofluorescence, and Western blotting were performed to evaluate α-SMA, vimentin, and p-ERK1/2 expression. *In vivo*, a PVR mouse model was established by intravitreal injection of ARPE-19 cells combined with platelet-rich plasma (PRP). Mice received a single intravitreal injection of luteolin (1 μL, 20 μM) or PBS on day 3, and retinal tissues were collected 28 days later for histological and protein analyses.

**Results:**

Our results revealed that luteolin, at concentrations of 12.5 μM and 25μM, significantly inhibited the horizontal and vertical migration ability of ARPE-19 cells. Luteolin, at concentrations of 12.5 μM and 25μM, also effectively inhibited the expression of EMT-related mesenchymal proteins induced by TGF-β2 in ARPE-19 cells. Luteolin inhibited intraocular mesenchymal proteins increase in experimental PVR in mice, followed by downregulation of p-ERK1/2 protein expression.

**Conclusion:**

Luteolin suppresses RPE cell migration and EMT both *in vitro* and *in vivo*, thereby alleviating PVR development. Its protective effect is associated with downregulation of ERK1/2 signaling. The inhibitory effects of luteolin suggest its potential as a therapeutic agent for PVR.

## Introduction

Proliferative vitreoretinopathy (PVR) is a serious complication following retinal detachment surgery. Essentially, it represents an abnormal wound healing response, involving various inflammatory and cytokine factors as well as the activation, migration, and proliferation of multiple cell types. This process leads to the formation of contractile fibrous membranes on both the anterior and posterior sides of the retina, ultimately exerting traction on the retina and causing retinal detachment (Ferro Desideri et al., 2024; Idrees et al., 2019).

PVR occurs in approximately 5%–10% of patients with rhegmatogenous retinal detachment and represents the most common cause of failed retinal reattachment. It is also a severe complication of ocular trauma, often resulting in profound and irreversible vision loss that greatly affects patients’ quality of life and mental wellbeing, imposing a significant socioeconomic burden (Quiram et al., 2006). Currently, surgery remains the primary treatment for PVR, aiming to reattach the detached retina and relieve traction from epiretinal and subretinal membranes. However, surgical intervention cannot suppress continuous cellular proliferation, leading to a high recurrence rate. Previous studies have reported that the anatomical success rate of PVR surgery ranges from 45% to 85%, whereas the functional success rate is only 26%–67%. This discrepancy is closely related to RPE layer irregularities, macular wrinkling, macular edema, and retinal fibrosis (Idrees et al., 2019; Assi et al., 2019).

To date, no effective pharmacologic therapy is available to prevent or halt PVR progression (Wang et al., 2024). Targeting RPE cell migration, proliferation, and EMT has therefore become a promising therapeutic approach. The extracellular signal-regulated kinase (ERK1/2) pathway has been identified as a key regulator of EMT and cell motility, and its activation has been reported in several ocular diseases, including diabetic retinopathy and retinal vein occlusion (Cao et al., 2024; Xu et al., 2025). TGF-β2 and extracellular signal-regulated kinase (ERK) play an important role in this process (Liu et al., 2024; Liu et al., 2021).

Luteolin (3′, 4′, 5,7-tetrahydroxyflavone) is a flavonoid available from a wide range of sources, with excellent antioxidant, anticancer, antibacterial, neuroprotective, cardioprotective, antiviral and anti-inflammatory properties (Huang et al., 2023; Prasher et al., 2022). In previous studies, luteolin was shown to have anti-proliferative effects on A549 lung cancer cells by reversing EMT through a mechanism involving contraction of the cytoskeleton, inducing expression of the epithelial marker protein E-cadherin, and downregulating the mesenchymal marker proteins N-cadherin, snail, and vimentin (Dai et al., 2020). EMT is also inhibited through inhibition of various signaling pathways (MAPK/AKT/PI3K pathway, NF-kB and STAT3) (Prasher et al., 2022). Recently, luteolin has been shown to be effective against various ocular diseases, including age-related macular degeneration, choroidal melanoma, and uveitis (Dongre and Weinberg, 2019; Shi et al., 2021). However, its role in PVR pathogenesis and its relationship with RPE cell EMT remain largely unexplored.

In this study, we investigated the effects of luteolin on PVR *in vitro* and *in vivo* and explored its potential mechanisms. We hypothesized that luteolin could attenuate PVR development by suppressing ERK1/2 signaling, thereby inhibiting EMT in RPE cells. To our knowledge, this is the first study to demonstrate the inhibitory effect of luteolin in a live PVR mouse model and to reveal a novel association between luteolin and the ERK1/2 pathway in RPE-mediated fibrosis. These findings provide new experimental evidence supporting luteolin as a potential antifibrotic candidate for ocular diseases.

## Materials and methods

### Cell culture and treatment

ARPE-19 cells (obtained from Guangzhou Saiku Biotechnology Co., Ltd.) were cultured in DMEM/F12 medium containing 1% penicillin-streptomycin and 10% inactivated fetal bovine serum (FBS). The cells were then placed in a cell culture incubator at 37 °C with a 5% CO2 atmosphere. After overnight incubation, the cells fully adhered to the wall. Subsequently, the cells were pre-treated with different concentrations (0, 6.25, 12.5, 25, 50 μM) of luteolin (Solarbio) for 48 h, and then starved overnight in serum-free medium. Subsequently, they were induced with 10 ng/mL of TGF-β2 in DMEM/F12 medium containing 1% FBS for an additional 48 h to induce EMT. Cell proliferation and migration were evaluated at different time points (0, 24, 48, and 72 h) after the cells reached a final density of 80%–90% (Chen et al., 2014; Chen et al., 2022).

### CCK-8 assay

When the cells reached 80%–90% confluence, they were enzymatically digested, collected, and centrifuged. The cell density was adjusted to 1 × 10^5^ cells/mL, and the cells were seeded in a 96-well plate. After 24 h, different concentrations of luteolin were added to the 96-well plates and incubated in a 37 °C, 5% CO2 incubator. After incubating for 0, 24, 48, or 72 h, the original culture medium was discarded, replaced with 10% CCK-8 reagent (40203ES80; Shanghai Yisheng Biotechnology Co., Ltd.), and incubated for 1‒4 h. The absorbance was measured at 450 nm using an enzyme marker (Thermo Fisher Scientific) (Zhang et al., 2023). This process was repeated three times with five replicates for each concentration.

### Cell scratch assay

Two lines were drawn on the back of the 6-well plate in advance; each line was the size of the well diameter to ensure that all subsequent photographs were taken in the same position. When the cells reached 80%–90% confluence, they were enzymatically digested, centrifuged, and collected. The cell density was adjusted to 1 × 10^7^/mL, and the cells were evenly seeded in a 6-well plate. Before the cells reached 80%–90% confluence, they were starved for 24 h and then marked with a 20–200 μL yellow gun tip perpendicular to the horizontal line on the back. Photographs were taken and recorded at fixed positions at 0, 12, 24, 36, and 48 h after marking. The migrated area was calculated using the following formula: (initial area - area at each time point)/initial area. Statistical analysis was performed to evaluate the differences in the migrated area over time (Shi et al., 2021). This process was repeated three times.

### Transwell migration assay

After treatment with different cell concentrations of luteolin, the cells were digested, centrifuged, and collected. We added 500–600 μL of prepared DMEM/F12 medium containing 10% fetal bovine serum to the lower chamber and 200 μL of cell suspension without serum to the upper chamber. Incubation was continued for 24 h. The following day, the cells were fixed with 4% paraformaldehyde for 30 min, stained outside the chamber with 0.1% crystal violet for 20 min, and counted. The migration of ARPE-19 cells was observed under an inverted microscope, randomly selecting five fields of view (including top, bottom, left, right, and center) with a ×20 objective lens, pictures taken, and the counts analyzed using ImageJ (Zhang et al., 2023). This process was repeated at least three times.

### Cell cycle was detected by flow cytometry

After sample processing, the cells were washed with PBS, fixed overnight in pre-cooled 75% ethanol. Subsequently, the cells were washed twice with PBS. For staining, 0.5 mL of PI staining solution was added to each sample. The cells were gently pipetted to ensure proper mixing, incubated for 15 min in the dark, and analyzed on a flow cytometry machine as soon as possible. The DNA content of the cells at each stage was determined using flow cytometry, and the resulting data were analyzed using FlowJo v10 software (Saika, 2006; Zhou et al., 2020). This process was repeated at least three times.

### Western blot

Proteins were extracted from ARPE-19 cells and mouse retinas, and the protein concentrations were determined using the BCA method. The experiment was performed with α-SMA (1:5000, Proteintech), vimentin (1:2000, CST), GAPDH (1:5000, Proteintech), p-ERK1/2 (1:2000, Immunoway), goat anti-rabbit antibody (1:5000, Boster), and goat anti-mouse antibody (1:5000, Boster). The target proteins were detected using an ECL chemiluminescence kit (Advansta). The blots were visualized using a Syngene G-Box Gel Imaging System (Liu et al., 2024; Xu et al., 2025).

### Cellular immunofluorescence

ARPE-19 cells were seeded in a 6-well plate and allowed to adhere for 48 h. Subsequently, the cells were washed with PBS, fixed with 4% paraformaldehyde for 15 min, permeabilized using 0.3‰ Triton X, and washed three times with PBS for 5 min each. The sections were blocked with 10% goat serum at room temperature for 1 h and incubated with vimentin (1:200, CST) at 4 °C overnight. The next day, the cells were washed three times with PBS for 5 min each and incubated with goat anti-rabbit antibody at room temperature in the dark for 1 h. After staining with DAPI for 5 min, an anti-fluorescence quencher was added, and the cells were observed under an inverted fluorescence microscope (Ferro Desideri et al., 2024). The entire process was repeated at least three times.

### Animal experiment

Male C57BL/6J mice aged 6–8 weeks were purchased from Nanjing Kerui Animal Co., Ltd. All experiments complied with the “ARVO Statement for the Use of Animals in Ophthalmic and Vision Research” and were approved by the Institutional Animal Care and Use Committee of Nanchang University (License No.: SYXK (Gan) 2021-0001). Twenty-four mice were divided equally into three groups: normal, ARPE-19 cells + platelet-rich plasma (PRP) + PBS, and ARPE-19 cells + PRP + Luteolin groups, all operated on in the right eye. Gatifloxacin eye drops was used 3 days before the operation. Mice were anesthetized via intraperitoneal injection of 3.6% concentration and 0.1 mL/10 g dose of chloral hydrate before the operation. The PVR model was established by the intravitreal injection of ARPE-19 cells and PRP using a 30G needle inserted through the sclera, 1 mm behind the corneal-scleral junction. Subsequently, 0.75 μL of ARPE-19 cells (approximately 1 × 10^5^/mL) and 0.75 μL of PRP were loaded into a 10 μL Hamilton syringe (Hamilton, Reno, NV), and the needle was removed to label the mice. Three days later, PBS or luteolin was injected into the vitreous cavities of the different groups. After 28 days, the PVR model was established, and the effects of luteolin on PVR in mice and the related molecular mechanisms were determined (Ferro Desideri et al., 2024; Hombrebueno et al., 2014; Pastor, 1998).

### Tissue immunofluorescence assay

The tablets were baked at 85 °C for 35 min and dewaxed with an environmental dewaxing solution for 30 min. They were then washed with a graded alcohol to water solution and rinsed with PBS for 10 min. To facilitate antigen retrieval, high-temperature repair was performed by immersing the tablets in an antigen retrieval solution for 8 min, followed by an 8-minute cooling period. This cycle was repeated once, and the tablets were then cooled to normal temperature. Next, permeabilization was achieved by treating the tablets with 0.5% Triton X-10 for 30 min. The eyeball was circled with a histochemical pen and blocked for more than 1 h. The vimentin protein was incubated overnight at 4 °C. The next day, the cells were incubated with goat anti-rabbit secondary antibody (1:200) for 1 h in the dark and was stained with DAPI for 5 min. Anti-fluorescence quencher was added to seal the slides, and the cells were observed and photographed under an inverted fluorescence microscope (Shi et al., 2021; Zhang et al., 2023). The entire process was repeated thrice.

### HE staining

The slices were baked in an oven at 85 °C for 35 min, dewaxed with the environmental dewaxing solution for 30 min, and rinsed with a graded alcohol to water. The sections were stained with hematoxylin for 1 min and then rinsed with distilled water (ddH2O). After staining with eosin for 30 s, the sections were rinsed again with ddH2O. To remove water marks, the slides were wiped in a dark box, focusing on the back and periphery of the slide. After thorough air-drying, the slides were sealed with neutral resin and a cover glass was placed over the eyeball area. The images were observed under an inverted microscope, and photographs were taken for documentation purposes (Zhang et al., 2023).

### Statistical analysis

The above experiments were repeated at least three times, and the data obtained were plotted and statistically analyzed using GraphPad Prism software (version 8.0). The data are presented as the mean ± SD. Statistical analysis was performed using ANOVA (analysis of variance) followed by Tukey’s *post hoc* test for multiple comparisons. A value of P < 0.05 was considered statistically significant (Buonato and Lazzara, 2014).

## Results

### Luteolin inhibits the proliferation of ARPE-19 cells

**FIGURE 1:**
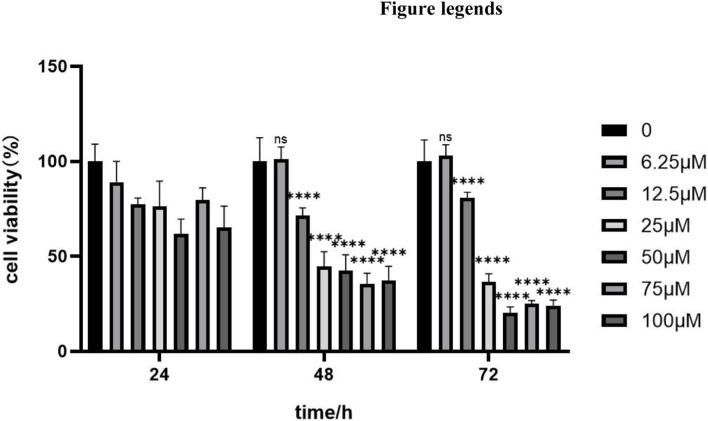
Luteolin inhibits the proliferation of ARPE-19 cells. ARPE-19 cells were treated with different concentrations of luteolin (0, 6.25, 12.5, 25, 50, 75, 100 μM LUT) for 24, 48, and 72 h. Compared with the control group, luteolin inhibited the proliferation of ARPE-19 cells in a dose- and time-dependent manner at 450 nm absorbance. nsP>0.05, ****P < 0.0001 vs. control group. Data are presented as mean ± standard deviation (n = 5/group).

**FIGURE 2:**
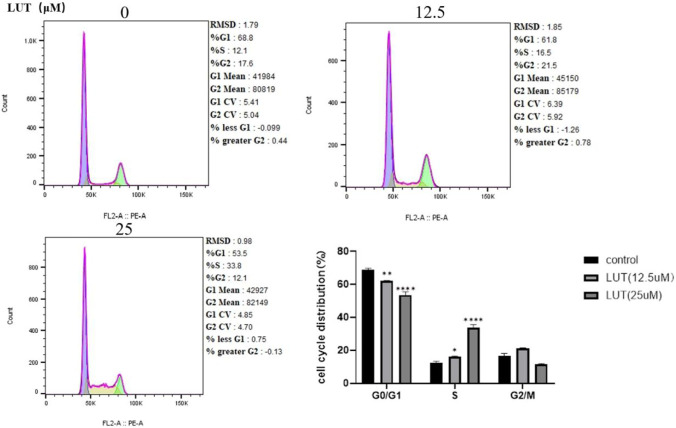
Luteolin modulates cell cycle progression and induces S-phase accumulation in ARPE-19 cells. ARPE-19 cells were treated with 12.5 μM and 25 μM luteolin for 48 h, and DNA staining was performed using propidium iodide (PI) for flow cytometry analysis. Compared with the untreated group, luteolin treatment increased the proportion of cells in the S phase in a concentration-dependent manner (*P < 0.05, **P < 0.01, ****P < 0.0001 vs. control group).

**FIGURE 3:**
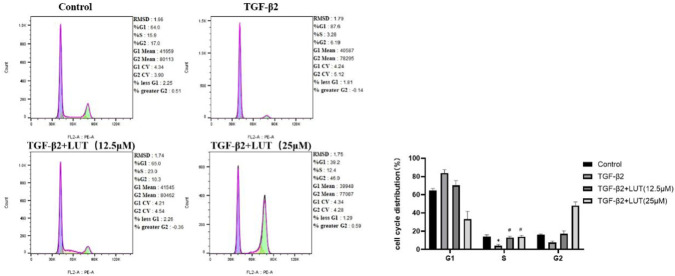
Luteolin modulates S-phase distribution and alleviates TGF-β2–induced cell cycle arrest in ARPE-19 cells. The number of S-phase cells increased in the 12.5 μM and 25 μM luteolin (LUT) groups compared with the TGF-β2 group, while there was no significant difference between the two concentrations (n = 3). Compared with TGF-β2 treatment alone, luteolin pretreatment partially restored the S-phase fraction. The cell cycle distribution is presented as mean ± standard deviation (*P < 0.05 vs. control group, #P < 0.05 vs. TGF-β2 group).

The OD value was measured using the CCK-8 assay, and the half-inhibitory concentration (IC50) of luteolin treatment for 48 h was calculated to be 37.54 μM. Among the tested concentrations, treatment with 25 μM luteolin for 48 h was identified as the optimal condition to achieve approximately 50% inhibition of cell viability without prolonging exposure. Cell viability decreased with increasing luteolin concentration and treatment time (Figure 1). To further analyze DNA cycle distribution, flow cytometry was performed on ARPE-19 cells treated with different concentrations of luteolin (0, 12.5, 25 μM) for 48 h. As luteolin concentration increased, the proportion of cells in G1 phase decreased while those in S phase increased (Figure 2). This suggests that luteolin interferes with normal cell cycle progression, leading to S-phase accumulation and reduced cell proliferation. When TGF-β2 was applied for 48 h, the number of G1-phase cells increased and S-phase cells decreased, consistent with EMT induction and cell cycle arrest. In contrast, in cells pretreated with luteolin, the proportion of G1-phase cells decreased while the S-phase fraction increased significantly (Figure 3). These findings indicate that luteolin mitigates TGF-β2–induced EMT in ARPE-19 cells, possibly by restoring normal cell cycle progression and maintaining proliferative balance.

### Luteolin inhibits the migration of ARPE-19 cells

**FIGURE 4:**
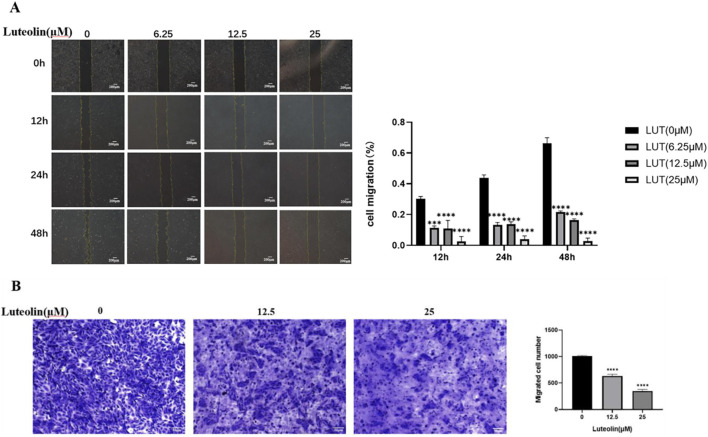
Luteolin inhibits the migration of ARPE-19 cells. ARPE-19 cells were treated with 0, 12.5 μM, and 25 μM luteolin for 12, 24, and 48 h. **(A)** The scratch assay was used to measure the horizontal migration of ARPE-19 cells at different time points. The histogram shows the mean ± standard deviation (n = 3). Scale bar: 200 μm. (***P < 0.001, ****P < 0.0001); **(B)** The Transwell assay was used to measure the vertical migration of ARPE-19 cells. The histogram shows the mean ± standard deviation of the migrated cells (n = 3). Scale bar: 50 μm. (****P < 0.0001).

Scratch and Transwell migration assays were used to assess the migration ability of ARPE-19 cells treated with different concentrations of luteolin. The scratch assay showed that untreated ARPE-19 cells had strong migration ability and almost completely closed the scratch after 48 h. However, after treatment with 6.25 μM, 12.5 μM, and 25 μM luteolin for 12 h, cell migration was inhibited. The cells’ migration ability did not show significant improvement with increased time (Figure 4A). The Transwell assay showed that after 24 h of treatment, the number of cells that migrated in the luteolin treatment group was significantly lower than that in the control group, and this effect was concentration-dependent (Figure 4B). The results from both assays confirmed that luteolin had a significant inhibitory effect on both the horizontal and vertical migration of ARPE-19 cells.

### Luteolin inhibits TGF-β2-induced EMT in ARPE-19 cells

**FIGURE 5:**
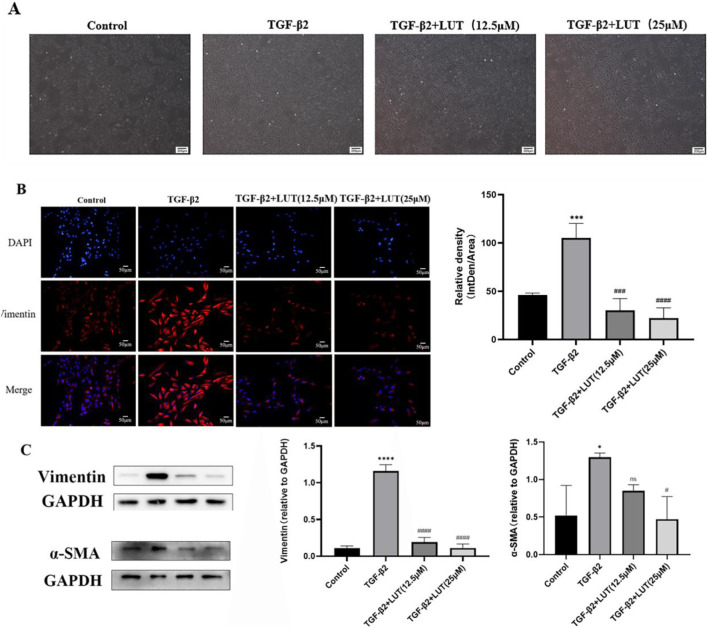
Luteolin inhibits TGF-β2-induced epithelial-mesenchymal transition in ARPE-19 cells. Cells were pretreated with or without luteolin for 48 h, followed by the addition of TGF-β2 for 48 h. **(A)** Cell morphology of cells treated with or without 12.5 μM, 25 μM luteolin, with or without TGF-β2 after 48 h. **(B)** Significant increase in fluorescence after 48 h of TGF-β2 treatment, an effect that can be inhibited by 12.5 μM and 25 μM luteolin. DAPI (blue), vimentin (red). Scale bar: 50 μM. **(C)** Protein expression was increased in the TGF-β2 group and significantly reduced in the group co-treated with 12.5 μM and 25 μM. (nsP>0.05, *P < 0.05, ****P < 0.0001 vs. control group; #P < 0.05, ##P < 0.001, ####P < 0.0001 vs. TGF-β2 group). All the above experiments were repeated at least three times.

In this study, TGF-β2-induced EMT was verified by evaluating cell morphology and the expression of mesenchymal-related proteins. The inhibitory effect of luteolin on this process was further examined by treating cells with different concentrations of luteolin. ARPE-19 cells, a human RPE cell line with the typical cobblestone-like morphology, underwent a marked morphological change after exposure to 10 ng/mL TGF-β2, showing elongated and spindle-shaped features. However, after luteolin treatment, cells gradually regained an epithelial-like appearance, with many cells recovering a hexagonal or cobblestone-like shape and only a few remaining elongated (Figure 5A). Western blot analysis revealed that α-SMA and vimentin protein levels were significantly increased after 48 h of TGF-β2 treatment, whereas 12.5 μM and 25 μM luteolin markedly reduced this upregulation in a concentration-dependent manner (Figure 5C). Immunofluorescence staining for vimentin showed strong fluorescence after TGF-β2 stimulation, indicating high expression compared with the control group. In contrast, pretreatment with 12.5 μM and 25 μM luteolin significantly reduced vimentin fluorescence intensity compared with the TGF-β2 group (Figure 5B). These findings confirm that TGF-β2 successfully induces EMT in ARPE-19 cells and that luteolin effectively suppresses this transition by downregulating mesenchymal markers such as vimentin and α-SMA.

### Luteolin inhibits TGF-β2-induced migration of ARPE-19 cells

**FIGURE 6:**
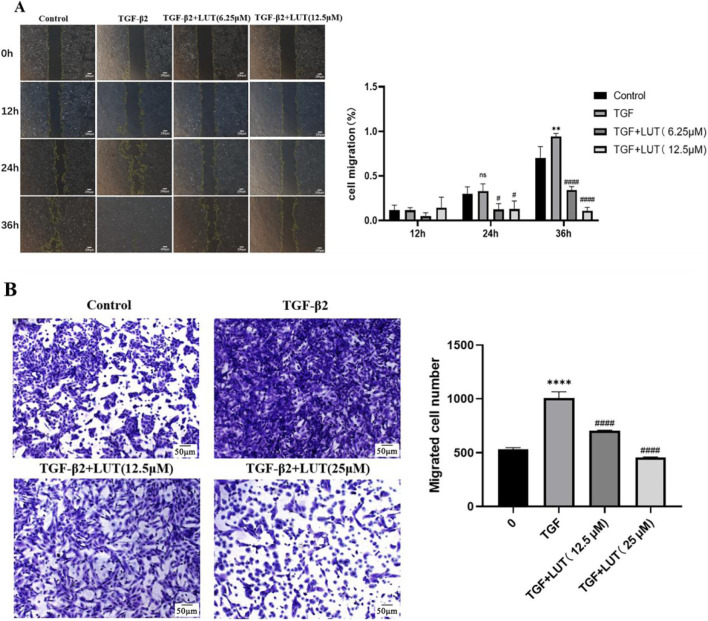
Luteolin inhibits the proliferation and migration of TGF-β2-induced ARPE-19 cells. After treatment with 0, 12.5μM, or 25 μM luteolin for 48 h, TGF-β2 was added or not added for another 48 h. **(A)** Scratch assay to measure the migration of ARPE-19 cells after epithelial-mesenchymal transition. Histograms were plotted with mean ± standard deviation (n = 3), scale bar: 200 μM. (nsP>0.05, **P < 0.01 vs. control group, ####P < 0.0001 vs. TGF-β2 group); **(B)** Transwell assay to measure the vertical migration of ARPE-19 cells. Histograms were plotted with mean ± standard deviation (n = 3), scale bar: 50 μM (****P < 0.0001 vs. control group, ####P < 0.0001 vs. TGF-β2 group).

As mentioned above, 10 ng/mL TGF-β2 successfully induced EMT. Scratch and transwell migration assays were used to study the effect of luteolin on the migration of TGF-β2-treated ARPE-19 cells. In the scratch assay, TGF-β2 alone promoted wound closure in a time-dependent manner. After pre-treatment with 6.25 µM or 12.5 µM luteolin, it inhibited the wound closure of TGF-β2-treated ARPE-19 cells (Figure 6A). In the Transwell assay, the TGF-β2 pre-treatment group promoted the vertical migration of ARPE-19 cells (P < 0.0001). Pre-treatment with different concentrations of luteolin before TGF-β2 treatment significantly reduced the migration ability of ARPE-19 cells, and the degree of inhibition increased with increasing luteolin concentration (P < 0.0001) (Figure 6B). These results indicated that luteolin inhibited the horizontal and vertical migration of TGF-β2-treated ARPE-19 cells.

### Effect of luteolin on the progression of proliferative vitreoretinopathy in mice

**FIGURE 7:**
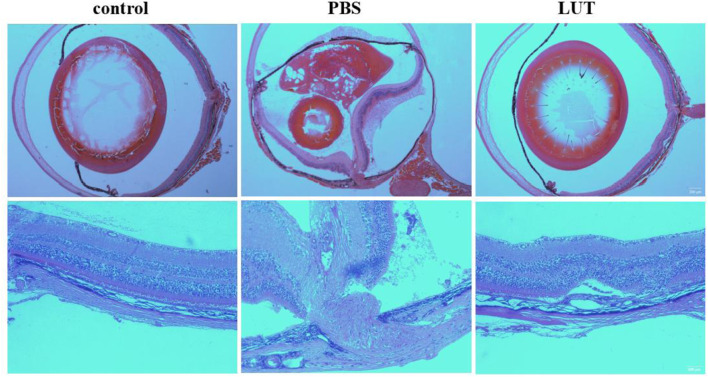
Hematoxylin and eosin staining of mouse eyeballs removed after 28 days. The normal mouse retina had a normal structure, with clear and orderly arranged layers. The retina in the PBS group was funnel-shaped and detached, with a large amount of proliferative membrane on the surface. The retina in the luteolin group had a normal structure, with only partial detachment. Scale bar: 200μm and 100 μm.

Starting from the day of induction (day 0), a slit-lamp examination was performed weekly to monitor the progression of PVR in the anterior segment and fundus of the mouse eye. Throughout the experiment, no accidental deaths, vitreous hemorrhages, cataracts, or infectious endophthalmitis were observed in any of the experimental animals. After 28 days of PVR induction, the eyeballs were removed, and paraffin sections were prepared and stained with hematoxylin and eosin (H&E). In the control group, the retinal layers were clear, the vitreous was transparent, the retinal structure was normal, and there were no signs of retinal folds or detachment points. Additionally, the vascular system was normal. Conversely, in the PBS group, the arrangement of the retinal cells was disrupted, the retina was funnel-shaped and detached, and the optic nerve was pulled with a large amount of traction membrane on the surface of the vitreous and retina, a large number of retinal folds, and swelling of the ganglion cell layer. In contrast, the luteolin group showed significant improvements in retinal morphology, with a significant reduction in the traction membrane on the surface of the vitreous and retina compared to the PBS group, and only partial folding and separation were observed (Figure 7).

**FIGURE 8:**
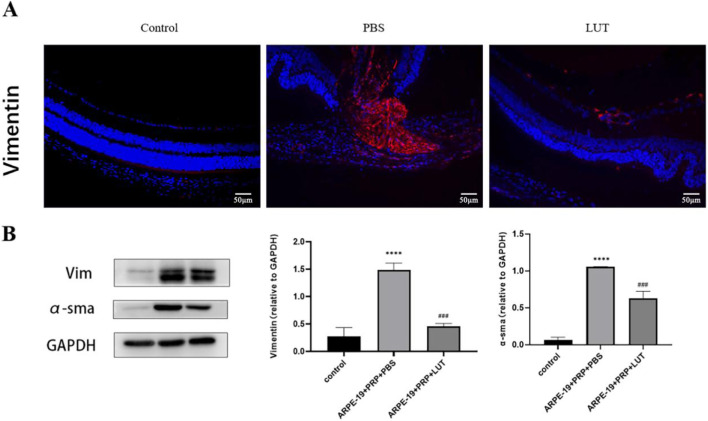
Luteolin inhibits the progression of proliferative vitreoretinopathy in mice. **(A)** Immunofluorescence staining showed that the fluorescence expression in the PBS group was significantly increased, while the expression in the luteolin group was decreased. Scale bar: 50 μM. **(B)** Western blotting showed that the protein expression of α-SMA and vimentin in the PBS group was increased, while the expression in the luteolin group was decreased. Histograms were plotted with mean ± standard deviation (n = 3). (****P < 0.0001 vs. control group; ###P < 0.001 vs. PBS group). The experiment was repeated three times.

At the same time, we further verified the results by performing immunofluorescence staining of mesenchymal proteins on paraffin sections and protein blotting experiments on mouse retinas. The immunofluorescence experiments showed that the fluorescence intensity of vimentin in the PBS group significantly increased, whereas that of vimentin in the luteolin group significantly decreased (Figure 8A). Western blot analysis showed that the protein expression of α-SMA and vimentin in the PBS group was significantly higher than that in the normal group (P < 0.0001), whereas the protein expression of α-SMA and vimentin in the luteolin group was significantly lower than that in the model group (P < 0.001) (Figure 8B). These results indicate that the PVR model, induced by injecting ARPE-19 cells combined with PRP into the vitreous cavity of mice, was successfully established and that luteolin injection into the vitreous cavity could reduce the severity of PVR.

### Luteolin inhibit the progression of PVR in mice through modulation of the ERK1/2 signaling pathway

**FIGURE 9:**
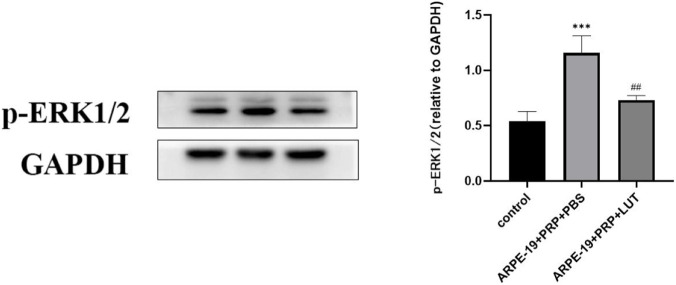
Luteolin modulates ERK1/2 pathway activation in a mouse model of proliferative vitreoretinopathy (PVR). Western blot analysis of phosphorylated ERK1/2 (p-ERK1/2) proteins in retinal tissues from control, PVR (model + PBS), and luteolin-treated groups. Compared with the control group, p-ERK1/2 expression was significantly increased in the PVR model group (P < 0.001), whereas luteolin treatment markedly reduced p-ERK1/2 levels compared with the model group (P < 0.01). Data are presented as mean ± SD (n = 3). *P < 0.05, **P < 0.01, ***P < 0.001 vs. control; #P < 0.05, ##P < 0.01 vs. model group.

The protein levels of phosphorylated ERK1/2 (p-ERK1/2) and total ERK1/2 were determined via Western blot analysis. Compared with the control group, the expression of p-ERK1/2 was markedly increased in the PBS-treated PVR model group (P < 0.001), while total ERK1/2 levels remained unchanged. In contrast, luteolin treatment significantly reduced the expression of p-ERK1/2 compared with the model group (P < 0.01), without affecting total ERK1/2 expression (Figure 9). These findings suggest that luteolin inhibit the progression of proliferative vitreoretinopathy (PVR) in mice by modulating ERK1/2 pathway activation rather than altering total protein expression.

## Discussion

PVR is a common ophthalmic disorder caused by dysregulated wound healing and represents one of the leading causes of blindness (Wang et al., 2021). Currently, surgical intervention remains the only treatment option; however, its efficacy is limited by recurrent retinal detachment. No pharmacological agent has yet been proven effective in preventing or treating PVR. Interventions can be approached from three major aspects: inflammation, cellular proliferation, and fibrosis (Pastor, 1998). In recent years, the antiproliferative, antimigratory, and epithelial–mesenchymal transition (EMT)–suppressive effects of luteolin in ocular diseases have attracted increasing attention. Shi et al. reported that luteolin inhibits the proliferation and migration of OCM-1 and C918 cells and elucidated its molecular mechanisms (Shi et al., 2021). Chen et al. found that flavonoids, including luteolin, dose-dependently reduce RPE cell proliferation, migration, and VEGF secretion (Chen et al., 2014).

Our experiments confirmed that luteolin can inhibit the proliferation, migration, and TGF-β2–induced EMT of ARPE-19 cells by blocking the S phase, while animal studies further demonstrated its ability to suppress the onset and progression of PVR by modulating ERK1/2 signaling (Chen et al., 2022; Zhang et al., 2023). Within the 6.25–25 μM range, luteolin significantly inhibited TGF-β2–induced proliferation, consistent with previous findings. Although luteolin treatment increased the proportion of G2/M-phase cells, this likely reflects a checkpoint arrest rather than enhanced proliferation, consistent with its antiproliferative effects (Xu et al., 2025; Zhou et al., 2020). At higher concentrations, however, the G2 fraction decreased, likely reflecting replication stress and increased apoptosis. Thus, moderate concentrations promote S→G2 arrest, whereas excessive levels may induce DNA damage. Further studies using EdU, Annexin V/PI, and γ-H2AX assays are needed to delineate these mechanisms.

The key step in PVR pathogenesis is the migration of quiescent RPE cells into the vitreous cavity and the formation of proliferative membranes on the retinal surface (Wang et al., 2024). Using scratch and Transwell assays, we confirmed that luteolin significantly inhibited TGF-β2–induced RPE migration. In agreement with prior research, TGF-β2 is abundant in the vitreous fluid of PVR patients, and its levels correlate with disease severity^[18.19]^. Western blot and immunofluorescence confirmed that TGF-β2 induces EMT in ARPE-19 cells, characterized by increased α-SMA and vimentin expression and a fibroblast-like morphology. Luteolin maintained epithelial morphology and reduced these mesenchymal markers, confirming its inhibitory effect on EMT. Although α-SMA immunofluorescence was not performed, Western blot analysis validated its protein changes, while vimentin staining supported the cytoskeletal transition typical of EMT. This limitation has been acknowledged and could be supplemented in future revisions if required.

Numerous signaling pathways are involved in EMT, including Smad, PI3K/AKT, TGF-β, Wnt, Notch, and ERK1/2 (Gonzalez and Medici, 2014; Vukelić et al., 2020). The ERK1/2 cascade, a major MAPK subfamily, plays a critical role in proliferation, migration, and fibrosis (Liu et al., 2021; Buonato and Lazzara, 2014; Liu and Kapron, 2010). Our study showed that p-ERK1/2 levels were elevated in PVR model mice but significantly reduced following luteolin administration. These results suggest that luteolin may attenuate PVR progression by modulating ERK1/2 signaling, providing a preclinical rationale for further investigation. However, this represents a correlative rather than causal relationship, and additional mechanistic studies using pathway activators or inhibitors (e.g., U0126) are warranted to clarify causality.

Although only a single dose of luteolin was tested, this was intended to verify mechanistic relevance rather than optimize dosage. The concentration was based on previous reports demonstrating ocular safety and antifibrotic activity (Zhang et al., 2023; Zhou et al., 2020). Moreover, while a sham-operated group was not included, earlier studies have shown that intravitreal PBS injection alone does not induce retinal fibrosis or gliosis (Hombrebueno et al., 2014; Heffer et al., 2020). Future research incorporating multiple doses and sham controls will improve reproducibility and translational reliability.

Clinically, luteolin’s low systemic bioavailability is well documented and may limit its effectiveness after oral or systemic administration. However, intravitreal delivery offers a distinct advantage by achieving high local concentrations while minimizing systemic exposure, making it a suitable route for posterior segment disorders like PVR. Future translational studies should assess luteolin’s intraocular pharmacokinetics, safety, and tissue distribution, and explore advanced sustained-release systems (e.g., nanoparticles, hydrogels, or intravitreal implants) to extend drug retention and reduce injection frequency. Compared with corticosteroids, anti-VEGF agents, or antifibrotic compounds such as mitomycin C and 5-fluorouracil (Heffer et al., 2020; Wang et al., 2024), luteolin’s multitarget properties—anti-inflammatory, antioxidant, and EMT-modulating—may offer broader therapeutic potential, though direct comparative studies are needed.

In summary, luteolin significantly inhibited TGF-β2–induced EMT in ARPE-19 cells and attenuated PVR progression *in vivo*. These findings suggest potential for further development and provide a preclinical rationale for exploring luteolin as an antifibrotic modulator in PVR. Nonetheless, given the limitations of single-cell-line and preliminary animal data, these conclusions should be interpreted within the context of early preclinical research. Future work on pharmacokinetics, ocular safety, delivery optimization, and comparative efficacy will be essential to determine whether luteolin can be translated into a viable therapeutic approach for PVR.

## Data Availability

The original contributions presented in the study are included in the article/supplementary material, further inquiries can be directed to the corresponding authors.
